# ngLOC: software and web server for predicting protein subcellular localization in prokaryotes and eukaryotes

**DOI:** 10.1186/1756-0500-5-351

**Published:** 2012-07-10

**Authors:** Brian R King, Suleyman Vural, Sanjit Pandey, Alex Barteau, Chittibabu Guda

**Affiliations:** 1 Department of Computer Science, Bucknell University, One Dent Drive, Lewisburg, PA, 17837, USA; 2 Department of Genetics, Cell Biology and Anatomy, University of Nebraska Medical Center, Omaha, NE 68198, USA; 3 Center for Bioinformatics and Systems Biology, University of Nebraska Medical Center, Omaha, NE 68198, USA

**Keywords:** Bayesian method, ngLOC, Protein subcellular localization prediction, *N*-gram-based approach, Protein sequence classification, Machine learning algorithm

## Abstract

**Background:**

Understanding protein subcellular localization is a necessary component toward understanding the overall function of a protein. Numerous computational methods have been published over the past decade, with varying degrees of success. Despite the large number of published methods in this area, only a small fraction of them are available for researchers to use in their own studies. Of those that are available, many are limited by predicting only a small number of organelles in the cell. Additionally, the majority of methods predict only a single location for a sequence, even though it is known that a large fraction of the proteins in eukaryotic species shuttle between locations to carry out their function.

**Findings:**

We present a software package and a web server for predicting the subcellular localization of protein sequences based on the ngLOC method. ngLOC is an *n*-gram-based Bayesian classifier that predicts subcellular localization of proteins both in prokaryotes and eukaryotes_._ The overall prediction accuracy varies from 89.8% to 91.4% across species. This program can predict 11 distinct locations each in plant and animal species. ngLOC also predicts 4 and 5 distinct locations on gram-positive and gram-negative bacterial datasets, respectively.

**Conclusions:**

ngLOC is a generic method that can be trained by data from a variety of species or classes for predicting protein subcellular localization. The standalone software is freely available for academic use under GNU GPL, and the ngLOC web server is also accessible at http://ngloc.unmc.edu.

## Findings

Protein subcellular localization prediction plays a crucial role in the automated function annotation of high-throughput studies. There are many computational methods that can predict protein subcellular localization [1,2]; yet, several limitations prevent their usage in proteome-wide prediction, including their inability to predict proteins localized to smaller or multiple organelles. Moreover, the majority of these tools are limited to predicting only a subset of organelles or a specific evolutionary species. We developed a probabilistic method called *ngLOC*, an *n*-gram based computational, machine-learned classification method that aims to address the majority of the stated limitations [3,4]. Specifically, ngLOC can predict a wide range of subcellular locations including multiple localizations of proteins, and it can be customized to work with a variety of datasets from prokaryotes to eukaryotes, including plant sequences. Moreover, ngLOC method makes its predictions solely based on the protein sequence information without the need for any extraneous information; hence, this method is highly favorable for proteome-wide prediction of subcellular localization.

Despite the number of methods that have been published for subcellular localization prediction, comparatively few tools are available to the research community in the form of standalone software or webservers [2,5]. Here, we present the first version of the ngLOC standalone software and an accompanying webserver for predicting subcellular localization of protein sequences from bacterial (gram-positive, gram-negative), plant and animal species. The web server, available at http://ngloc.unmc.edu, provides an intuitive, user-friendly interface for generating predictions for a given set of sequences. The standalone software is released with complete source code, training datasets and a user manual.

### Data collection

We developed four different training datasets for this new release of the ngLOC method. All datasets consist of curated set of protein sequences taken from the Swiss-Prot database release as of May 17th 2011 [6] that contains experimentally determined annotations on subcellular localization. Sequences were gathered and assembled into four distinct datasets based on the species of evolutionary origin. Plant sequences were obtained from the species that fall under division Streptophyta (mostly land plants) of the kingdom Viridiplantae, and animal sequences were obtained from species that fall under kingdom, Metazoa. Likewise, two prokaryotic datasets were assembled from bacteria under Gram-negative or Gram-positive categories. Additionally, we have applied the following filters to obtain high-quality data for testing and training our program: (i) sequences with predicted or ambiguous localizations were removed, (ii) sequences shorter than 10 residues in length were removed, (iii) all redundant sequences were removed, and (iv) annotations of sequences known to localize in multiple locations were manually checked for accuracy. The location-wise distributions of our datasets for eukaryotic and prokaryotic species are shown in Tables 1 and 2, respectively.

**Table 1:** Eukaryotic training datasets

**Localization class**	**Code**	**Animal**	**Plant**
Cytoplasm	CYT	2513	481
Cytoskeleton	CSK	778	550
Endoplasmic Reticulum	END	870	121
Extracellular/Secreted	EXC	9618	238
Golgi Apparatus	GOL	290	59
Lysosome	LYS	215	---
Mitochondria	MIT	2348	469
Nuclear	NUC	4216	630
Plasma Membrane	PLA	6006	351
Peroxisome	POX	183	50
Cell Junction	JNC	62	---
Chloroplast	CHL	---	4862
Vacuole	VAC	---	131
Multiple localizations		3309	304
**TOTAL**		**30318**	**8246**

**Table 2:** Prokaryotic training datasets

**Localization class**	**Code**	**Gram-negative**	**Gram-positive**
Cytoplasm	CYT	4139	1776
Extracellular	EXC	263	292
Inner Membrane	IN	1397	347
Outer Membrane	OUT	344	---
Periplasm	PER	415	---
Cell Wall	WAL	---	32
**TOTAL**		**6558**	**2447**

## Implementation

### Standalone software package

The ngLOC software package is developed entirely in C++ using the GNU gcc framework, version 4.2. A detailed user manual is provided in the package and also separately, on the ngLOC web server to help understand how to configure and execute the method during installation. The program can be downloaded and installed in four quick steps as described in the ‘ReadMe.txt’ file. The downloadable package also comes with training datasets derived from different evolutionary species as outlined in Tables 1 and 2. The user manual leads the user through a basic analysis using the training datasets from animal species. ngLOC program offers a rich set of options in the configuration file (config.ini) to alter the *n*-gram size, prediction score thresholds, input and output formats, etc. More advanced settings such as altering the species group, and/or the number and type of subcellular codes to be predicted can be done in the definitions file (defs.h). The entire source code, licensed under the GNU General Public License (see http://www.gnu.org/copyleft/gpl.html for complete details) and the training datasets are supplied with the package to enable further development and integration with other high-throughput data analysis pipelines. As we have noted in prior studies [3], if researchers are interested in developing their own training datasets, they will need to carefully consider the optimal value of *n* for the *n*-gram model. It is strongly dependent on the size of the dataset, and the measure of similarity in the dataset.

### ngLOC Web Server

A web-based interface for predicting the subcellular localization of the user-supplied protein sequence(s) is available at http://ngloc.unmc.edu/. The interface is simple to use, and is designed to predict the top three most probable subcellular localizations for any given protein sequence using the ngLOC method. To generate predictions, protein sequences must be supplied in the FASTA format. Sequences can be provided in the text window of the browser or a file containing a set of sequences (maximum file size of 10MB) can be uploaded from the local machine. Since the prediction model varies by the evolutionary species, the user must select the appropriate species grouping from the pull-down menu before starting the prediction. There are four groupings of species to choose from: (i) *Animal*, (ii) *Plant*, (iii) *Gram-positive bacteria* and (iv) *Gram-negative bacteria*. The Animal species group will be selected by default. The type of subcellular localizations predicted will strongly depend on this selection. For example, if the *Animal* species is chosen, the program will never predict the localization of a sequence as chloroplast, which is an option only under plant species.

**Figure 1:**
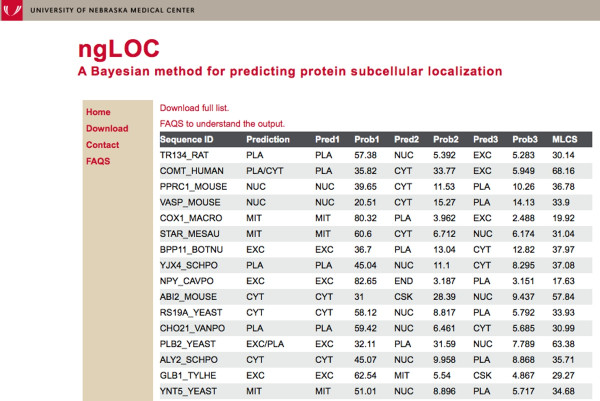
Details of the query output showing the top three predictions with probability scores.

The web version of ngLOC uses a file read mechanism to access the pre-built ngLOC model rather than creating a new model every time a search is performed; thus the queries run much faster. A regular search with up to a 100 sequences takes no more than 45 seconds, while a 10MB file upload containing about 20,000 sequences may take about 5 minutes. The output format includes the top three predicted locations along with associated confidence scores for each class (Figure 1). Additionally, the MLCS (Multi-Localization Confidence Score) is also reported, which reflects if the top two locations are predicted within a close probability margin [3]. If the MLCS equals or exceeds 60.0, the prediction column in the output shows the top two predictions separated by a ‘/’ character. For instance, sequences that shuttle between cytoplasm and nucleus can be predicted as ‘CYT/NUC’.

## Results and discussion

We use a Naïve Bayesian classification method to model the density distributions of fixed-length peptide sequences (*n*-grams) over each distinct subcellular location (for more information please refer to King and Guda, 2007 [3]). These distributions are determined from protein sequence training datasets (Table 1 and 2) that contain experimentally determined annotations of subcellular localizations. This program can predict 11 distinct locations each in plant and animal species. ngLOC also predicts 4 and 5 distinct locations on gram-positive and gram-negative bacterial datasets, respectively. Using leave-one-out validation, we report standard performance measures over each subcellular location. For the animal predictive model, an *n*-gram value of 7 was used for the n-gram model, whereas plant and bacterial models were induced using an *n*-gram value of 6. An exhaustive discussion behind the choice of the ideal value of *n* is included in the original paper [3]. Results for the latest datasets on animal and plant data are displayed in Table 3, and for the bacterial data in Table 4. The overall prediction accuracy varies from 89.78% to 91.4% across species.

**Table 3:** Class-wise performance of ngLOC method on eukaryotic datasets

		**Animal**				**Plant**			
**Localization class**	**Code**	**Prec.**	**Sens.**	**Spec.**	**MCC**	**Prec.**	**Sens.**	**Spec.**	**MCC**
Cytoplasm	CYT	0.818	0.750	0.983	0.762	0.864	0.832	0.991	0.838
Cytoskeleton	CSK	0.937	0.784	0.998	0.853	0.988	0.965	1.000	0.976
Endoplasmic Reticulum	END	0.970	0.785	0.999	0.869	0.876	0.645	0.999	0.748
Extracellular	EXC	0.953	0.946	0.974	0.922	0.966	0.723	0.999	0.831
Golgi Apparatus	GOL	0.940	0.593	1.000	0.745	1.000	0.509	1.000	0.712
Lysosome	LYS	0.949	0.693	1.000	0.810				
Mitochondria	MIT	0.979	0.852	0.998	0.906	0.912	0.727	0.995	0.804
Nuclear	NUC	0.805	0.914	0.960	0.831	0.769	0.873	0.976	0.802
Plasma Membrane	PLA	0.876	0.957	0.961	0.890	0.796	0.866	0.989	0.822
Peroxisome	POX	0.946	0.760	1.000	0.847	0.906	0.580	1.000	0.724
Cell Junction	JNC	0.774	0.387	1.000	0.547				
Chloroplast	CHL					0.946	0.977	0.899	0.889
Vacuole	VAC					0.844	0.702	0.998	0.766
**% Overall accuracy**					**89.88%**				**91.39%**

Prec-precision; Sens-sensitivity; Spec-specificity; MCC-Matthews correlation coefficient.

**Table 4:** Class-wise performance of ngLOC method on prokaryotic datasets

		**Gram-Negative**				**Gram-Positive**			
**Localization class**	**Code**	**Prec.**	**Sens.**	**Spec.**	**MCC**	**Prec.**	**Sens.**	**Spec.**	**MCC**
Cytoplasm	CYT	0.887	0.992	0.785	0.822	0.888	0.993	0.668	0.755
Extracellular	EXC	0.940	0.597	0.998	0.742	0.899	0.637	0.990	0.731
Inner Membrane	IN	0.908	0.830	0.977	0.835	0.941	0.648	0.993	0.754
Outer Membrane	OUT	0.987	0.680	1.000	0.812				
Periplasm	PER	0.925	0.561	0.997	0.707				
Cell Wall	WAL					0.786	0.344	0.999	0.516
**% Overall accuracy**					**89.78%**				**89.33 %**

Prec-precision; Sens-sensitivity; Spec-specificity; MCC-Matthews correlation coefficient.

### Benchmarking the confidence score

Our results displayed in Table 3 and Table 4 are based on including all predictions for every sequence in the dataset using the leave-one-out validation method. As a probabilistic method, every prediction is generated with an estimated probability of correctness. This is an important criterion to consider when studying the results generated by the ngLOC method. Table 5 displays the accuracy of the predictions based on confidence score (CS) for both the plant and animal data. These results clearly demonstrate the value of the CS in evaluating the reliability of a prediction. Predictions with a high CS score have a high accuracy rate and vice-versa. For example, a prediction in the *Animal* model that attained a score of 70 or higher has a 99.9% likelihood of being correct. Moreover, 65.8% of the entire dataset tested received a score at these levels. On the contrary, if a prediction only scored less than 20, it has only about a 50% chance of being the correct prediction; only 5% of the data was scored at this low confidence level. The cumulative accuracy in Table 5 reflects the coverage of prediction at a given CS.

**Table 5:** - Benchmarking the confidence score on eukaryotic datasets

		**Confidence Score**									
		**0**	**10**	**20**	**30**	**40**	**50**	**60**	**70**	**80**	**90**
Animal	% of dataset	-	5.0	10.5	5.5	5.4	5.4	7.8	11.2	21.0	28.3
	% accuracy	-	50.2	48.9	80.4	89.0	95.4	98.4	98.9	99.5	99.9
	Cumulative % of data	100.0	100.0	95.0	84.5	79.0	73.6	68.2	60.5	49.3	28.3
	Cumulative % accuracy	89.9	89.9	92.0	97.3	98.5	99.2	99.5	99.6	99.8	99.9
Plant	% of dataset	-	7.9	6.1	4.6	4.8	4.7	6.1	8.3	14.3	43.2
	% accuracy	-	40.3	65.8	83.9	88.8	94.1	97.8	99.0	99.9	100.0
	Cumulative % of data	100.0	100.0	92.1	86.0	81.4	76.6	71.9	65.8	57.5	43.2
	Cumulative % accuracy	91.4	91.4	95.8	97.9	98.7	99.3	99.7	99.9	100.0	100.0

### Comparison against other methods

We compared the updated ngLOC method against two recent methods. Our first comparison was against SherLoc2, a method that can predict 11 eukaryotic subcellular localizations [6]. SherLoc2 integrates several sequence-based features, text-based features, phylogenetic profiles and Gene Ontology (GO) terms to generate a prediction. Our second comparison was against WegoLoc, which predicts 10 eukaryotic subcellular localizations of proteins based on sequence similarity and weighted Gene Ontology (GO) information [7]. Both methods support predictions for plant and animal sequences.

We created two separate datasets for testing purposes. We generated a random selection of approximately 15% of our training data for animal and plant data, respectively. Sequences in the test set were removed from the training set for these experiments. We also removed all instances in the test data that also existed in the training data for WegoLoc. (We were unable to obtain the training data for sherLoc2.) Sequences belonging to cell junction were removed. All other test data were considered, including multi-localized sequences. For multi-localized sequences, we consider the prediction to be correct for all methods if *any* of the correct localization classes were predicted.

The results from comparing ngLOC against sherLoc2 are displayed in Table 6 and 7. A local, stand-alone version of sherLoc2 was installed to run our tests. We encountered numerous sequences that failed to report a result from sherLoc2 (this was particularly true of the plant test), and thus we only include classes and data on the proteins that generated a prediction. Our results show that the ngLOC method outperformed sherLoc2 in most classes with superior accuracy. This is likely due to the fact that sherLoc2 requires data from multiple sources, including text sources, to develop seven different classifiers joined together to generate a single prediction. Some of these individual classifiers scan for known localization signals, motifs, phylogenetic profiles, known GO terms, and text abstracts from PubMed [6]. If this information is not available for sequences being predicted, then we observed that in many instances, an incorrect prediction was generated. In contrast, our ngLOC method is a sequence-only, homology-based classification method that has no need for additional information a priori.

**Table 6:** Class-wise performance of ngLOC and SherLoc2 on animal test dataset

	**Localization**	**ngLOC (TP)**	**ngLOC (sens.)**	**sherLoc2 (TP)**	**sherLoc2 (sens.)**	**Total**
	CYT	212	78.5	238	**88.1**	270
	CSK	60	**72.3**	0	0.0	83
	END	72	**78.3**	52	56.5	92
	EXC	930	**95.1**	508	51.9	978
	GOL	12	**60.0**	3	15.0	20
	LYS	6	**60.0**	2	20.0	10
	MIT	185	**86.4**	112	52.3	214
	NUC	357	**91.1**	149	38.0	392
	PLA	556	**95.0**	275	47.0	585
	POX	14	87.5	14	87.5	16
Total (Single)		2404	**90.4**	1353	50.9	2660
Total (Multi)		249	**82.5**	218	72.2	302
TOTAL		2653	**89.6**	1571	53.0	2962

Bold letters denote better performance. TP- true positives; sens- sensitivity. Class-wise sensitivities are calculated for single-localized sequences only.

**Table 7:** Class-wise performance of ngLOC and SherLoc2 on plant test dataset (single-localized only)

**Localization**	**ngLOC (TP)**	**ngLOC (sens.)**	**SherLoc2 (TP)**	**SherLoc2 (sens.)**	**Total**
CYT	45	84.9	49	**96.2**	53
END	13	**76.5**	9	58.8	17
GOL	4	**40.0**	3	30.0	10
CSK	13	**100.0**	**0**	0.0	13
MIT	40	69.0	36	**74.1**	58
NUC	63	**78.8**	46	72.5	80
PLA	36	**90.0**	1	5.0	40
EXC	21	**67.7**	14	61.3	31
CHL	539	**99.3**	**68**	0.0	543
VAC	10	**66.7**	**1**	0.0	15
POX	3	**60.0**	2	40.0	5
TOTAL	787	**91.0**	229	26.5	865

Bold letters denote better performance. TP- true positives; sens- sensitivity.

Our second test evaluated ngLOC against predictions from WegoLoc [7]. The results are displayed in Table 8 and 9. All tests were conducted on the WegoLoc web server. We chose the animal or plant training dataset from Hoglund (selectable on the server) for our tests as appropriate [8]. The WegoLoc method utilizes a variety of external tools and sources to generate a prediction, including the use of BLAST to find the most similar sequence, and then applying the full set of GO annotations from UniProtKB that are associated with the data. Specifically, it weights the GO terms according to its association with subcellular localization. On the majority of classes in the animal test, the WegoLoc method performed well against ngLOC; this was expected due to the amount of information being used a priori. However, it did not handle any proteins localized to the cytoskeleton correctly, nor did it do well with plasma membrane proteins. Additionally, ngLOC outperformed WegoLoc on multi-localized data. The ngLOC method surpassed the WegoLoc method on overall accuracy, with a final result of 89.3% vs. 87.8% of the data in the test set predicted correctly on animal data. The ngLOC method performed better on the plant test, where ngLOC outperformed WegoLoc in the majority of classes. Our overall accuracy yielded 91.0% vs. 56.9% for WegoLoc. Part of this is due to the lack of any correct predictions for cytoskeleton. Another significant contributor to its poor performance is due to the large number of proteins localized to the chloroplast, where they yielded a sensitivity of 49.5% compared to our 99.2%. This is probably due to lack of many GO annotations for plant data as there are for animal data. For additional studies and comparisons that were performed against other datasets and methods, please refer to our original publication [3].

**Table 8:** Class-wise performance of ngLOC and WegoLoc on animal test dataset

**Localization**	**ngLOC (TP)**	**ngLOC (sens.)**	**WegoLoc (TP)**	**WegoLoc (sens.)**	**Total**	
CSK	93	**79.5**	0	0.0	117	
CYT	271	75.1	329	**91.1**	361	
END	90	76.9	93	**79.5**	117	
EXC	1298	94.7	1307	**95.3**	1371	
GOL	18	58.1	19	**61.3**	31	
LYS	22	81.5	23	**85.2**	27	
MIT	301	84.3	344	**96.4**	357	
NUC	555	90.8	581	**95.1**	611	
PLA	798	**96.4**	689	83.2	828	
POX	16	66.7	24	**100.0**	24	
Total (Single)	3462	**89.8**	3409	88.5	3854	
Total (Multi)	415	**85.4**	400	82.3	486	
**TOTAL**	3877	**89.3**	3809	87.8	4340	

Bold letters denote better performance. TP- true positives; sens- sensitivity. Class-wise sensitivities are calculated for single-localized sequences only.

**Table 9:** Class-wise performance of ngLOC and WegoLoc on plant test dataset (single-localized data only)

	**ngLOC (TP)**	**ngLOC (sens.)**	**WegoLoc (TP)**	**WegoLoc (sens.)**	**Total**
CYT	54	81.8	63	**95.5**	66
END	9	**69.2**	5	38.5	13
GOL	2	22.2	4	**44.4**	9
CSK	22	**95.7**	0	0.0	23
MIT	39	75.0	50	**96.2**	52
NUC	53	80.3	60	**90.9**	66
PLA	35	**79.5**	19	43.2	44
EXC	20	**66.7**	14	46.7	30
CHL	587	**99.2**	293	49.5	592
VAC	11	61.1	12	**66.7**	18
POX	4	**66.7**	3	50.0	6
TOTAL	836	**91.0**	523	56.9	919

Bold letters denote better performance. TP- true positives; sens- sensitivity.

The ngLOC method has several distinctive advantages over existing methods, especially for making genome-wide predictions. Since the method is solely sequence based, preparation of training and testing datasets is easier and the method can be broadly applicable without the need for additional annotation data for making predictions. Moreover, despite our comparison against two other methods, both of which require additional information beyond sequence, ngLOC still performed well. Second, designing a pure probabilistic model yields many benefits: (i) a proven confidence score based on the probability generated is output with each prediction, allowing the researcher to utilize only high-confidence predictions; (ii) the probability measure is used to generate a separate score that can estimate the likelihood of a given sequence being multi-localized; (iii) a probabilistic model allows one to investigate the internal dependent features of the model (i.e. our n-grams) that are correlated to certain class, leading to a wide range of interesting studies, such as the investigation of novel targeting signals. Finally, this method performs particularly well in predicting proteins from smaller organelles like Golgi, lysosomes, peroxisomes, etc. [3,9], which are typically difficult to predict by other methods.

## Applications of this method

The ngLOC method is a Bayesian classification method that was developed to predict the subcellular localization of new protein sequence data. This method is capable of predicting the localization of proteins to all the major and minor locations in all species. In particular, this method is designed to work with genome-scale data for predicting the entire subcellular proteomes [3]. Our current work has focused on two major areas: (1) broadening the coverage of the method through incorporating support for different species, including Animal, Plant, and Gram-positive and Gram-negative bacteria; and (2) development of a downloadable source code and corresponding web server to make this method available to the research community. The web server provides a readily available resource to get immediate predictions for tens of thousands of protein sequences. The entire source code and training data are available to allow local installation of this software for subcellular localization prediction to be conducted on any computer platform. The local installation version facilitates its integration with genome-scale data analysis pipelines. ngLOC is a generic classification method at its core. Though we have developed the method specifically for subcellular localization, other uses of the model are starting to surface. For example, in a recent study, similar *n*-gram based methods were applied for detecting biological language models [10]. With minor modifications to the source and configuration files, it can be extended to classify protein sequences that are labeled with a wide range of classifications, with a potential to go beyond subcellular localization.

### Availability and requirements

**Project name**: ngLOC – A Bayesian method for prediction protein subcellular localization.

**Project home page**: http://ngloc.unmc.edu.

**Operating system(s)**: Windows, Linux, Mac OS-X.

**Programming language**: C++.

**Other requirements**: N/A.

**License**: GNU GPL.

**Any restrictions to use by non-academics**: N/A.

## Abbreviations

MLCS: Multi-Localized Confidence Score; GNU: GNU’s Not Unix (a recursive acronym); GPL: General Public License.

## Authors’ contribution

CG conceived the original study, generated the datasets and provided overall framework for this project. BK developed the method and original software. SV and SP worked on the web-based system. AB worked on the user manual and standalone software installation.

## Author information

BRK (Assistant professor) has a strong background in computer science and mathematics. He developed the original ngLOC method with CG. SV is a graduate student in CG’s group with training in computer science. SP is a bioinformatics programmer in CG’s group with training in computer science and web/database development. AB is an undergraduate student in BRK’s group who is getting training in computer science. CG (Associate professor) has an interdisciplinary background both in molecular and computational biology. He has published a number of computational prediction methods on protein subcellular localization since 2004.

## Competing interests

None.
